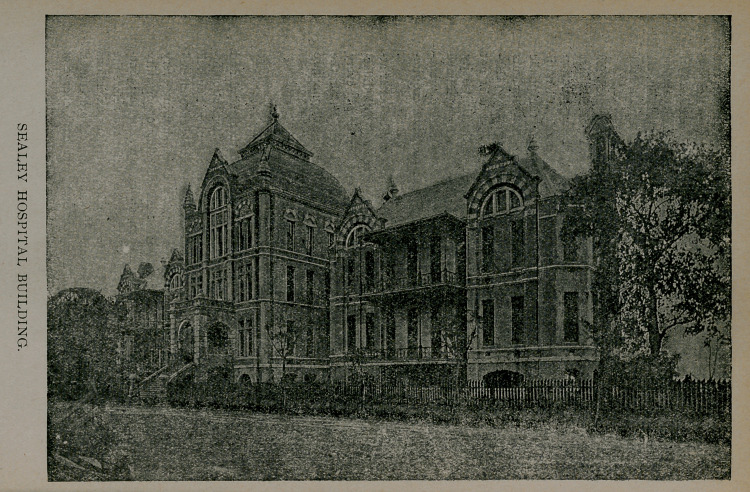# The Texas Medical College

**Published:** 1892-07

**Authors:** 


### MEDICAL DEPARTMENT UNIVERSITY OF TEXAS.

More than a quarter of a century has passed since the people of this country laid down their arms and returned to the pursuits of peace. In that time Texas has more than doubled her population, and has developed a wealth of resources that has astonished the world. Schools and churches have sprung up all over its vast plains, and she feeds now from the bosom of her soil near on to three millions of people. And yet, only at this late date has she established a school of medicine, while smaller and poorer States have them almost by the score. This seems remarkable, too, in view of the fact that Texas furnishes more medical students, with one exception, than any State in the Union; material enough, if controlled, to support ten schools. According to Dr. Jno. H. Ranch, late Secretary Illinois State Board of Health, the number of medical students from Texas in 1891 in attendance in the various colleges throughout the United States was four hundred and ninety-three. This rich patronage should be utilized at home; the vast amount of money these young men spend abroad would support home schools in comfort.

The University of Texas was inaugurated in Austin in 1881. By vote of the people the Medical Department was located at Galveston, on account, no doubt, of the advantages of anatomical and clinical material, there being several hospitals in Galvesveston and none in Austin.

Not untll 1891, however, was the medical branch inaugurated. There were difficulties innumerable to be overcome, but finally, by donations of Galveston and appropriations by the legislature, both the elegant college building and the grand hospital, pictured herewith, were erected, and opened October 1st, 1891, for medical teaching. The State pays the professors liberal salaries, which overcomes one of the greatest evils connected with the subject of medical teaching; it makes them independent of any income from classes, and consequently enables them to fix the standard of requirements high. It is not, like some other really able schools, compelled to have a large class, and obliged to graduate a number of students to insure its own existence.

Consequently the Board of Regents, of which Dr. Thos. D. Wooten is President, established a high standard of requirements, and the Faculty arranged a thorough curriculum of

THE TEXAS MEDICAL COLLEGE BUILDING.

study, divided into three courses; and beginning October 1st, 1891, the first course of lectures and clinics was given the past winter to a class of twenty-four, graduating three in April, 1892. The first diplomas were issued to and the degree conferred upon Messrs. H. T. Guinn, J. P. Hendricks and Thomas Flavin, on the 21st of April, 1892, at the first annual commencement, held at the Opera House, in Galveston.

This apparently discouragingly small class is accounted for by the Honorable Dean, Prof. J. F. Y. Paine, M. D., in his first report to the Board of Regents, as follows: "The explanation is found in the fact that the Faculty had not been selected until a short time before the opening of the session, and the public announcement of the first course was so delayed that students had either already left the State or arranged plans to go elsewhere. Even now, letters of inquiry indicate that it is not generally known that the Medical Department of the University is in operation. Again, a certain proportion of young men have been kept away for the reason their insufficient literary preparation would not stand the test of our matriculation requirement."

There is not a doubt that the second course will be better attended. The College is well equipped for teaching, both didactically and clinically, ample facilities for demonstration in every department being supplied. Prof. Seth M. Morris, M. D., the professor of chemistry, is now in Berlin, where he will purchase the latest laboratory apparatus for his department.

Anatomical material is abundant, and

THE SEALEY HOSPITAL

affords splendid facilities for clinical study, both medical and surgical. Last session there were treated in this institution fifteen hundred patients. Of this number, about nine hundred were medical cases, embracing every disease usually encountered in this latitude, and six hundred surgical cases, showing every form of injury and surgical disease, and affording opportunity for every operation generally met in an ordinary surgical practice. Sixty-five surgical operations were performed before the class, embracing amputations, laparotomies, resections, excissions of tumors, and in fact all the more important surgical procedures. The Faculty constitute the Hospital Staff, and have charge of that institution.

## Figures and Tables

**Figure f1:**
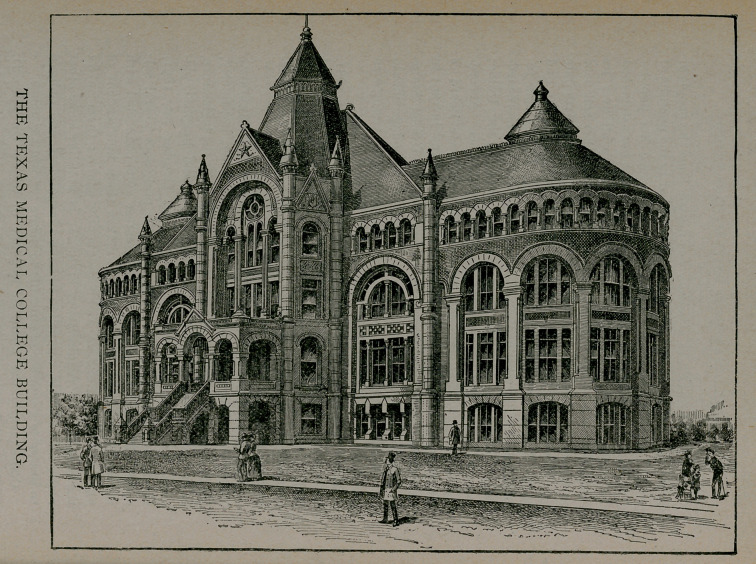


**Figure f2:**